# Supplementary material for: Gamma-Synuclein Dysfunction Causes Autoantibody Formation in Glaucoma Patients and Dysregulation of Intraocular Pressure in Mice
Source: Biomedicines. 2022 Dec 27;11(1):60. doi: 10.3390/biomedicines11010060 (PMC9856171; doi:10.3390/biomedicines11010060)
Supplement: Supplementary file 1 [file biomedicines-11-00060-s001.zip › biomedicines-2108177-supplementary.pdf]

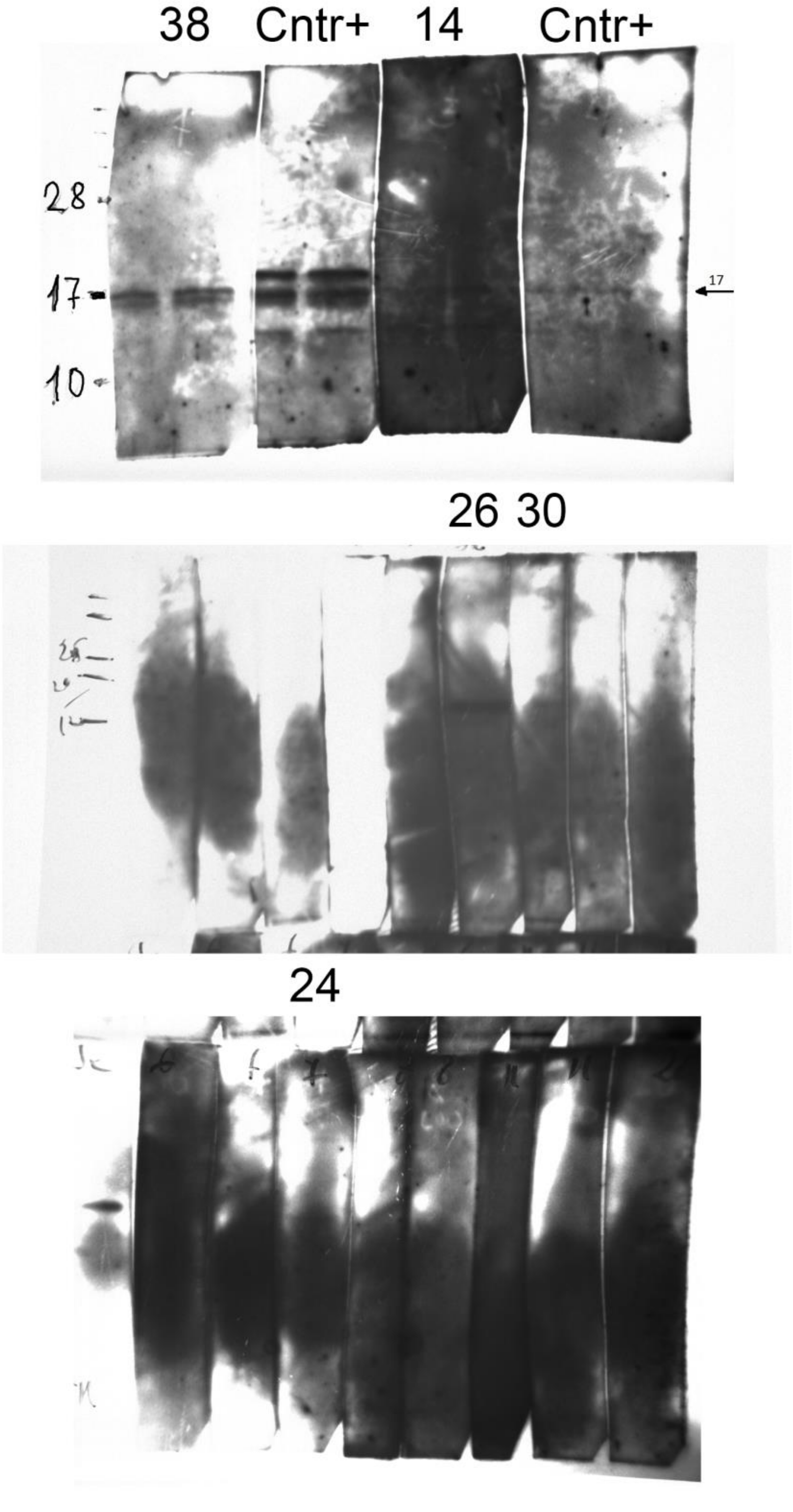

Figure S1: Original, unmodified images of X-ray films for Western blots. Numbers indicate all positive serum samples. Cntr+ sample – positive serum from our previous work.
